# Transient splenial lesion syndrome in bipolar-II disorder: a case report highlighting reversible brain changes during hypomanic episodes

**DOI:** 10.3389/fpsyt.2023.1219592

**Published:** 2023-07-10

**Authors:** Jingyuan Zhou, Yuan Cao, Gaoju Deng, Jinbo Fang, Changjian Qiu

**Affiliations:** ^1^Mental Health Center, West China Hospital of Sichuan University, Sichuan Clinical Medical Research Center for Mental Disorders, Chengdu, China; ^2^Department of Nuclear Medicine, West China Hospital of Sichuan University, Chengdu, China; ^3^Department of Psychiatry and Psychotherapy, Jena University Hospital, Jena, Germany; ^4^West China School of Nursing, West China Hospital of Sichuan University, Chengdu, China

**Keywords:** reversible splenial lesion syndrome, hypomanic episodes, case report, bipolar disorder type 2, corpus callosum

## Abstract

**Background:**

Reversible splenial lesion syndrome (RESLES) is a rare neurological condition characterized by temporary abnormalities in the splenium of the corpus callosum, which has been reported in mental disorders. Previous studies on bipolar disorder (BD) primarily focused on aspects such as brain structure and function, neurochemical changes, and genetics. However, there have been no studies reporting the occurrence of this syndrome during hypomanic episodes and its disappearance during the remission phase in bipolar disorder type 2 (BD-II).

**Case presentation:**

We present a case report of a 30 years-old female patient with BD-II who exhibited symptoms of RESLES during a hypomanic episode. The patient, with a 12 years psychiatric history, has experienced recurrent depressive episodes initially, with the first hypomanic episode occurring 8 years ago. During this period, this patient made several visits to the outpatient clinic to have her medications adjusted due to repeated suicide attempts. This time, she was admitted to our hospital with a second hypomanic episode due to drug withdrawal during pregnancy. The RESLES was observed on her brain magnetic resonance image, and it was alleviated after treatment with lithium carbonate and quetiapine until achieving remission.

**Conclusion:**

We present the first report of identifying RESLES in BD-II with hypomanic episodes, which subsequently disappears during the remission phase. Our case report highlights a potential association between BD and RESLES, emphasizing the need for future studies to explore the underlying mechanisms connecting these two conditions in greater depth.

## Introduction

Reversible splenial lesion syndrome (RESLES) is a clinical radiological syndrome characterized by the presence of a focal lesion often involving the central area of the splenium of the corpus callosum (SCC). The most common neurological symptoms of this syndrome include headache, dizziness, seizure, disturbance of consciousness, and mental abnormality (1, 2). Generally, these symptoms are reversible in most cases, and patients typically recover in a few days or weeks. The estimated prevalence of RESLES was approximately 3% (3), however, as not all patients with clinical manifestations undergo magnetic resonance image (MRI), this figure is likely to be an underestimate.

The RESLES can be caused by various factors such as infections, metabolic disturbances, and medications. It has been reported in several diseases including viral encephalitis, influenza, epilepsy, and psychiatric disorders (2, 4, 5). Studies have reported the RESLES in mental disorders including major depressive disorder (MDD) and bipolar disorder (BD). Xu et al. (6) suggested that RESLES might only show mental symptoms and in their research, all patients showed different mental disorders as the only manifestation with different diagnosis (MDD, dissociative and conversion disorders, undifferentiated somatoform disorder, unspecified psychosis and BD). A recent case reported two patients of RESLES with lithium-associated neurotoxicity while they were diagnosis as schizophrenia and BD, respectively (7). Although the underlying pathophysiology of RESLES is not yet well understood, studies have indicated that it is related to inflammation, intramyelinic cytotoxic edema (8), oxidative stress (9), and autoimmune processes (10). Vega-Nunez et al. (11) mentioned in their review that there is a certain association between inflammatory biomarkers and mood episode, such as tumor necrosis factor alpha (TNF-α), C-reactive protein (CRP), cytokines, etc. Serum levels of these inflammatory biomarkers may increase or decrease with different mood episode. Meanwhile, another study (12) suggests that psychiatric disorders may have the potential to function as inducers of a wide range of immune responses, leading to a dysregulation of the inflammatory status and the cell-mediated immunity. Therefore, we speculate that these inflammatory responses may link RESLES and BD from a pathophysiological perspective.

BD is a serious chronic disease characterized by the alternate occurrence of manic and depressive symptoms. The survey revealed a 12 months prevalence rate of 1.5% and a lifetime prevalence rate of 2.4% for BD (13). Although the exact mechanism of BD remains unknown, it is believed that a combination of genetics, environment, and alterations in brain structure and chemistry may contribute to its development (14). Previous studies observed a gray matter and white matter volume reduction in cingulate area in BD patients (15). Besides, the functional MRI indicted functional alterations in striatal regions, as well as functional connectivity reduction between the postcentral gyrus, the precentral gyrus, middle frontal gyrus and other brain regions in BD patients compared with healthy controls (16, 17). Studies suggest that alterations in the tryptophan-kynurenine pathway can lead to an imbalance in the production of inflammatory molecules. This dysregulation of the pathway can result in increased inflammation in the central nervous system, contributing to the development and progression of BD. In turn, chronic inflammation can further disrupt the normal functioning of neurotransmitters and neuronal circuits, exacerbating the symptoms of BD (18, 19).

However, previous literature only reported RESLES in different diseases, and just focused on the occurrence of RESLES and its potential causes at a certain time in different diseases, little attention has been given to such a case that RESLES presents in bipolar disorder type 2 (BD-II) with hypomanic episodes and disappears in the remission phase. We followed up on a case of a female BD-II patient who developed the RESLES during a hypomanic episode and found that this sign disappeared after her condition resolved. That provided the necessity of the present case.

## Case presentation

We reported a 30 years-old female patient with BD-II, who began with depressive episodes in her previous 12 years history. During the course of antidepressant treatment, she showed hypomanic manifestations such as high mood, increased speech, and subsequently adjusted her medication several times in the outpatient clinic. This time, she was hospitalized for hypomania again after drug withdrawal because of her pregnancy. No abnormalities were found in her physical examination at admission, and the blood sampling results were also normal. Only RELSLES was found in MRI, and it was alleviated after treatment with lithium carbonate and quetiapine until achieving remission.

This patient with a psychiatric history of recurrent depressive episodes since the early 4 years of her whole course of the disease (12 years), with the typical melancholic presentation, has a first hypomanic episode at 22 years old, under treatment with Venlafaxine while discontinued magnesium valproate. During this period, the patient exhibited prominent clinical symptoms including elevated mood, increased activity, heightened self-confidence, decreased need for sleep, excessive spending on unnecessary items, engaging in a new romantic relationship and engaging in sexual activity with the new partner despite already being in a committed relationship. The phases lasted almost 2 months. After that, she was diagnosed with bipolar disorder and received magnesium valproate and quetiapine for treatment. In the second half of the same year, the patient was admitted to the outpatient hospital unit repeatedly for modifying pharmaco-therapy after repeated suicide attempts by overdose, slitting her wrists with a knife, electrocuting, etc. During this period, the patient’s mood gradually stabilized. After 8 years, the patient was admitted to our hospital for a second hypomanic episode due to drug withdrawal while she was pregnant.

**Figure 1:**
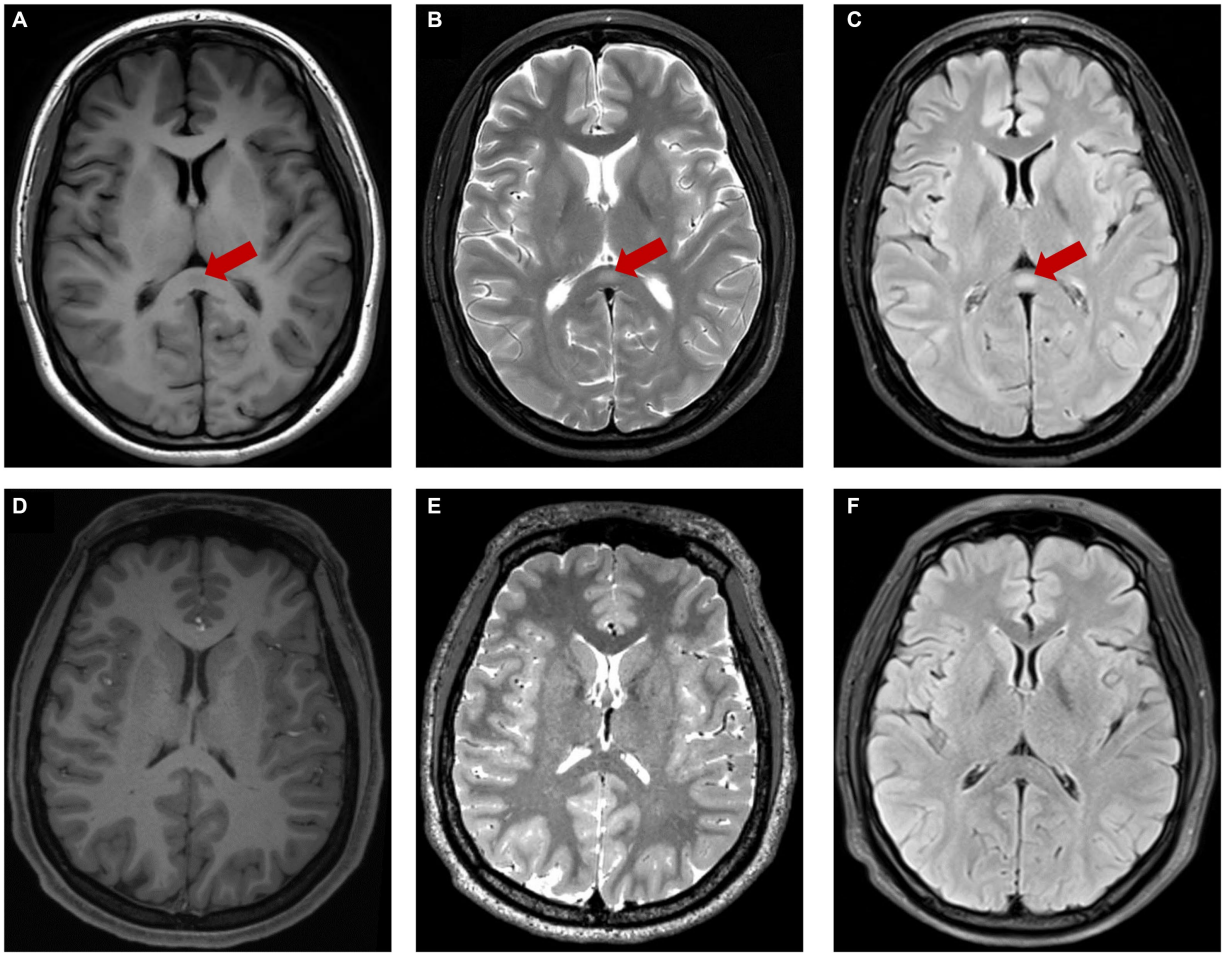
The red arrow showed lesion center on SCC characterized by hypo-intensity on T1WI **(A)**, hyper-intensity on T2WI **(B)**, T2-FLAIR **(C)**. The lesion was disappeared after treatment **(D–F)**.

During the hospitalization, her physical examination showed no abnormality and the psychiatric evaluation showed hypomanic presentation such as increased language volume and speech rate, faster thinking, decreased need for sleep, irritability, mood swings, excessive energy. This patient denied any related family history. Laboratory blood examination findings were normal. The patient scored 4 points on the young mania rating scale (YMRS) and 21 points on the hypomania symptom checklist (HCL-32). The MRI revealed an isolated, well-circumscribed lesion in the splenium of the corpus callosum. The lesion appeared hyperintense on T2 weighted image (T2WI) and T2 FLAIR sequence, isointense to slightly hypointense on T1weighted image (T1WI) (Figures 1A–C). After 2 weeks of treatment with lithium carbonate and quetiapine, her mood was gradually stable, and clinical and functional symptom got improved.

Two months after discharge, she was in clinical remission of BD and the followed-up MRI showed that the abnormal signals in the splenium of the corpus callosum had completely disappeared (Figures 1D–F).

## Discussion

Here we reported a 30 years-old female BD-II patient in a hypomanic episode with reversible splenial lesion syndrome and the RESLES was disappeared when she was stable. BD is a common and recurrent disease that seriously affects the social and life functions of patients, however, the specific neurophysiologic basis of that is still unknown.

The corpus callosum, also named Neocortical Commissure, is the largest white-matter bundle that connects the bilateral cerebral hemispheres and plays an important role in interhemispheric communication and coordination (20). It can be divided into four parts: the rostrum, the genu, the body, and the splenium. To date, extensive research has begun to focus on observing changes in the corpus callosum in patients with BD (21). In general, BD patients presented alterations in the volume and structure of the corpus callosum, which may be accompanied by a decline in the function of the corpus callosum accordingly. As an important structure connecting the left and right hemispheres of the brain, damage to the corpus callosum may affect the functional coordination between the two hemispheres, resulting in a series of clinical symptoms. Previous studies showed that BD adults demonstrated a globally thinner and smaller callosum, and the most frequently or seriously affected area is the splenium, midbody (22–24). Additionally, studies have demonstrated the corpus callosum, particularly the splenium is more vulnerable to cytotoxic edema than other brain areas (25). In light of a remarkable model concerning emotional regulation made by Shobe and his colleagues (26), the right hemisphere mediates the identification and comprehension of emotional stimuli, and the left hemisphere shares and processes the emotional information transmitted from the right hemisphere via the corpus callosum. Any microstructural abnormalities in the callosum may degrade the exchange of information between the hemispheres. Yasuno et al. (27) fo*und that* at least one function of the corpus callosum is a strict determinant of the IFC. Its impairment could disrupt functional integration and coordination between the hemispheres.

The brain MRI of our patient showed that the splenium of the corpus callosum had a slightly hypointense signal on T1-weighted imaging and a hyperintense signal on T2WI and T2-FLAIR sequence, which may suggest the presence of cytotoxic edema (25, 28). In previous studies, Bellani et al. (20) and Yang et al. (21) impairment of the corpus callosum in patients with BD is associated with disruptions of the integrity of white matter, such as localized edema, astrogliosis, and demyelination. What we found in this patient also confirms the possible neurophysiologic basis mentioned above.

At the same time, earlier we mentioned that edema may lead to RESLES, while the pathophysiology of RESLES is obscure. It was reported in a wide spectrum of clinical conditions that RESLES have been found in association with many conditions, such as drug therapy, malignancy, infection, trauma, metabolic abnormalities, and other entities (29), commonly seizures and antiepileptic drugs (30). The clinical presentation is nonspecific, the symptoms were relieved after treatment of the etiology, and the prognosis was good. This is the first time RESLES has been identified during hypomanic episodes in BD patients who have stopped taking drugs.

Thus, we conjecture that RESLES and hypomanic episodes may be connected with some kind of cytotoxic edema in the splenium, according to a report, this condition can occur with abrupt changes in serum level, particularly upon withdrawal (4). Meanwhile, Yoshinobu Takahashi and colleagues considered that this edema can occur within an uncomplicated pregnancy or after delivery, which may be secondary to reversible vasoconstriction (31). According to Tanaka et al. (18, 19, 32), the tryptophan-kynurenine metabolic pathway may further provide some evidence for this conjecture. The abnormality of the kynurenine pathway may cause mental illness, and the mental illness will in turn act on the kynurenine pathway to further aggravate this abnormality (33), thus producing a variety of metabolites. These metabolites may promote an inflammatory response that results in the central nervous system being in a chronic inflammatory state, further increasing the risk of cell edema. With the advancement of treatment, the patient’s condition gradually stabilized, and the lesions also disappeared in remission.

However, RESLES, as a self-limiting disease, can be controlled by the body’s own immune response and gradually recover. Previous studies have not explicitly linked RESLES and BD from a pathophysiological perspective, so the existence of a mechanistic association between these two diseases is still a hypothesis. Perhaps in future studies, we will be able to explore more potential connections between these two diseases.

## Conclusion

In conclusion, we described the first report of finding RESLES in BD-II with hypomanic episodes and disappearing in the remission phase. The shortcoming of our case is that we did not collect a specimen of cerebrospinal fluid from the patient to provide additional data support. In fact, whether the treatment can be improved by looking for such similarities, and whether the prognosis means that patients with BD comorbidity RESLES can recover better. The answers to these questions are still unknown. We hypothesize that the pathogenesis of RESLES may have some similarities to the hypomanic episode of BD-II. However, based on current research alone, we do not yet know whether RESLES, as a self-limiting disorder, is actually associated with the hypomanic episode of BD-II, or whether the co-occurrence of these two diseases is merely a coincidence. Thus, providing more research data to support the connection between these two diseases may be the next research goal. Our case report sheds light on a potential link between BD-II and RESLES. Further investigations are warranted to elucidate the mechanisms underlying this connection and expand our understanding of the relationship between these two entities.

## Data availability statement

The original contributions presented in the study are included in the article/supplementary material, further inquiries can be directed to the corresponding authors.

## Ethics statement

Written informed consent was obtained from the individual(s) for the publication of any potentially identifiable images or data included in this article.

## Author contributions

JZ wrote the first draft of the paper. YC, CQ, and JF edited the paper. CQ supervised the study conduction. JZ, YC, GD, and JF were involved in clinical data collection. All authors contributed to the article and approved the submitted version.

## Funding

This study was supported by the Department of Science and Technology of Sichuan provincial government (Grant No. 2022YFS0345).

## Conflict of interest

The authors declare that the research was conducted in the absence of any commercial or financial relationships that could be construed as a potential conflict of interest.

## Publisher’s note

All claims expressed in this article are solely those of the authors and do not necessarily represent those of their affiliated organizations, or those of the publisher, the editors and the reviewers. Any product that may be evaluated in this article, or claim that may be made by its manufacturer, is not guaranteed or endorsed by the publisher.
